# Direct anterior versus posteriorlateral approachs for clinical outcomes after total hip arthroplasty in the treatment of severe DDH

**DOI:** 10.1186/s12891-022-05759-y

**Published:** 2022-11-05

**Authors:** Yiping Lan, Eryou Feng, Bei Li, Zhiming Lu, Feitai Lin, Yan Weng

**Affiliations:** 1 The Third Clinical Medical College, Fujian Medical University, No. 47, Shangteng Road, Cangshan District, Fuzhou, China; 2 Fujian University of Traditional Chinese Medicine, Fuzhou, China

**Keywords:** Direct anterior approach, Posterolateral approach, Severe developmental dysplasia of the hip, Clinical efficacy, Prosthesis placement parameters

## Abstract

**Background:**

The total hip arthroplasty (THA) has gained popularity in in the treatment of severe developmental dysplasia of the hip (DDH). the posterior lateral approach (PLA) has good clinical efficacy and has been confirmed by the majority clinicians. Nevertheless, controversy exists regarding longer-term benefits of the direct anterior approach (DAA). The objective of this study was to investigate the clinical efficacy and placement of S-ROM prosthesis in the treatment of severe DDH by The total hip arthroplasty (THA) with different surgical approaches.

**Methods:**

A retrospective analysis was performed on 42 patients with severe DDH admitted to our hospital from August 2015 to February 2022, who were treated with S-ROM prosthesis for total hip arthroplasty and subtrochanteric osteotomy of the femur. They were divided into DAA group and PLA group according to different surgical approaches. Perioperative indicators and imaging data were collected.

**Results:**

The surgery time, intraoperative blood loss, and creatine kinase difference in DAA group and PLA group was without a statistically significant difference (*P* > 0.05). The postoperative length of hospitalization was shorter in the DAA group than in the PLA group (6.50 ± 3.15 vs 9.18 ± 4.93, *P* = 0.045). The acetabular abduction angles、the acetabular anteversion angles, the safe area ratio, The difference of femoral eccentricity, and the vertical difference of rotation center in DAA group and PLA group, there was no statistical significance (*P* > 0.05). Statistically significant differences were detected the horizontal difference of rotation center (*P* = 0.044).

**Conclusions:**

Total hip arthroplasty with S-ROM prosthesis is a feasible procedure for severe dysplastic DDH. The clinical efficacy and prosthesis placement parameters of DAA approach are advantage to those of PLA approach.

**Supplementary Information:**

The online version contains supplementary material available at 10.1186/s12891-022-05759-y.

## Introduction

Developmental dysplasia of the hip is a general term for a group of pathologies characterized by spatial and temporal instability of the hip joint during development, ranging from mild acetabular dysplasia without hip dislocation to high hip dislocation, which is mainly manifested by abnormal acetabular and femoral morphology [1, 2]. Total hip arthroplasty has been reported to be effective in improving pain, correcting lower limb length, and restoring joint function in DDH [3–5]. However, as per Crowe’s diagnostic criteria [6], type III and IV are the most severe, and most patients with these types experience deformities, such as high-position false acetabulum, true acetabulum bone defect, excessive femoral neck anteversion, and femoral stem deformation, leading to long operation time, slow postoperative rehabilitation, and numerous complications, which is a real challenge for THA. restoration of the anatomical hip center in DDH will cause limb lengthening, which may lead to potential complications such as sciatic nerve palsy and arterial injury. Additionally, some studies have shown that the selection of different prosthesis materials during THA will can result in metal deposition such as blood high high cobalt (Co), chromium (Cr) and molybdenum (Mo) after THA [7, 8]. The clinical outcome of THA is afflicted by the positioning of the acetabulum and femoral implants, the selection of prosthesis, and the application of osteotomy. The modular femoral stem prosthesis S-ROM, designed specifically for this purpose, is an ideal choice for cases requiring subtrochanteric osteotomy of the femur because it can provide maximum corrective force through distal and proximal press-fit fixation [9, 10] and assume a critical role in correcting femoral anteversion angle and in anti-rotational stabilization during osteotomy. Therefore, S-ROM has become the best choice for the use of prosthesis in THA for patients with high-dislocated DDH.

Traditional surgical approaches for THA comprise the posterior lateral approach, whose efficacy has been proven in the treatment of Crowe III or IV DDH by a wide range of clinical clinicians. The direct anterior approach is a true surgical approach between the neural interface and the muscle gap [11], which has the advantages of less damage to soft tissues, less bleeding, no contraindicated postoperative position, and quick recovery [12]. Currently, there are few reports on the treatment of Crowe III or IV DDH through DAA, and no research has been conducted to compare the postoperative clinical outcomes of THA via DAA and PLA in patients with severely high-dislocated DDH. This study set out to retrospectively analyze the clinical efficacy of the S-ROM prosthesis in the treatment of Crowe III or IV DDH through different approaches of DAA and PLA.

## Materials and methods

### General information

Inclusion criteria were as follows: (1) the surgical side met the diagnostic criteria of congenital Crowe type III or IV DDH, with typical clinical manifestations and X-ray manifestations; (2) patients received initial THA through DAA or PLA; (3) S-ROM femoral stem prosthesis was applied; (4) imaging data were available;

Exclusion criteria were as follows: (1) cases underwent revision; (2) patients suffered from Crowe type I and II DDH; (3) patients had incomplete follow-up data; (4) patients had a history of previous hip surgery; (5) patients experienced the deformation of the acetabulum and femoral head on the non-surgical side of the hip due to advanced osteoarthritis, femoral head necrosis, and other diseases; (6) patients suffered from comorbidities of diseases (e.g., polio and Parkinson’s Disease) that severely affected postoperative rehabilitation.

This study was a retrospective analysis. Our study enrolled patients with Crowe III or IV DDH who underwent initial THA using the S-ROM prosthesis (Johnson & Johnson, New Brunswick, New Jersey, USA) between August 2015 and February 2022 from the database of our Department of Orthopaedics. The hip prosthesis used for surgery was a Johnson & Johnson product from the United States. All surgeries were performed by a senior surgeon. A total of 42 patients were included, with an average follow-up time of 2.38 years. According to the surgical approach.

### Preoperative preparation

THA 2D digital planning was performed Preoperatively. patients routinely underwent X-rays of both lower limbs, anteroposterior X-ray of bilateral hip joints, lateral X-ray of affected hip joints, anteroposterior and lateral plain X-ray of lumbar vertebra, and computed tomography (CT) specialist examinations. The height of dislocation, the bone length of lower limbs, acetabular rotation centers, pelvic inclination, and lumbar scoliosis were evaluated to decide whether to conduct osteotomy and to select a suitable prosthesis before surgery.

### Surgical techniques

#### DAA for THA and subtrochanteric osteotomy of the femur

The patient lied in a supine position. The DAA incision was chosen to extend 2-3 cm upward and 2 cm distally compared to the conventional incision which began 2 cm distal and 3 cm lateral to the bilateral anterior superior iliac spine, pointing to the fibular head. The skin, subcutaneous tissues, and fascia were incised layer by layer, the Tensor fascia lata muscle fibers and sarcolemma were bluntly separated to enter the Heuter space was entered. Then, the joint capsule was exposed by loosening part of caput reflexum musculi recti femoris and the fat and connective tissues in front of the joint capsule. The joint capsule was incised in an “L” or “inverted T” shape.

The femoral neck was amputated vertically at 0.5-1.0 cm above the lesser trochanter to obtain the femoral head. The joint capsule was excised and the acetabular labrum and scar tissues were cleaned from the false acetabulum downward. The triangular-shaped dysplastic true acetabulum was found at the superior margin of the obturator to evaluate the bone quality of the anterior and posterior walls of the true acetabulum. The acetabulum was reconstructed using a 36 mm acetabular file with a number-by-number filing at a depth bounded by the internal plate, maintaining an abduction angle of 40° ± 5° and an anteversion angle of between 15° and 20°. The morphology of the bone defect was observed to identify the need for bone grafting and the mode of bone grafting [13]. If the acetabular cup bone coverage was > 70%, a small cup prosthesis was placed directly into the true acetabulum, whereas if the bone coverage was < 70%, the intercepted femoral head was trimmed, and then a structural bone graft was made above the posterior acetabulum. The acetabular prosthesis was fitted, screws were entered, and a polyethylene inner cushion was fitted.

**Fig.1:**
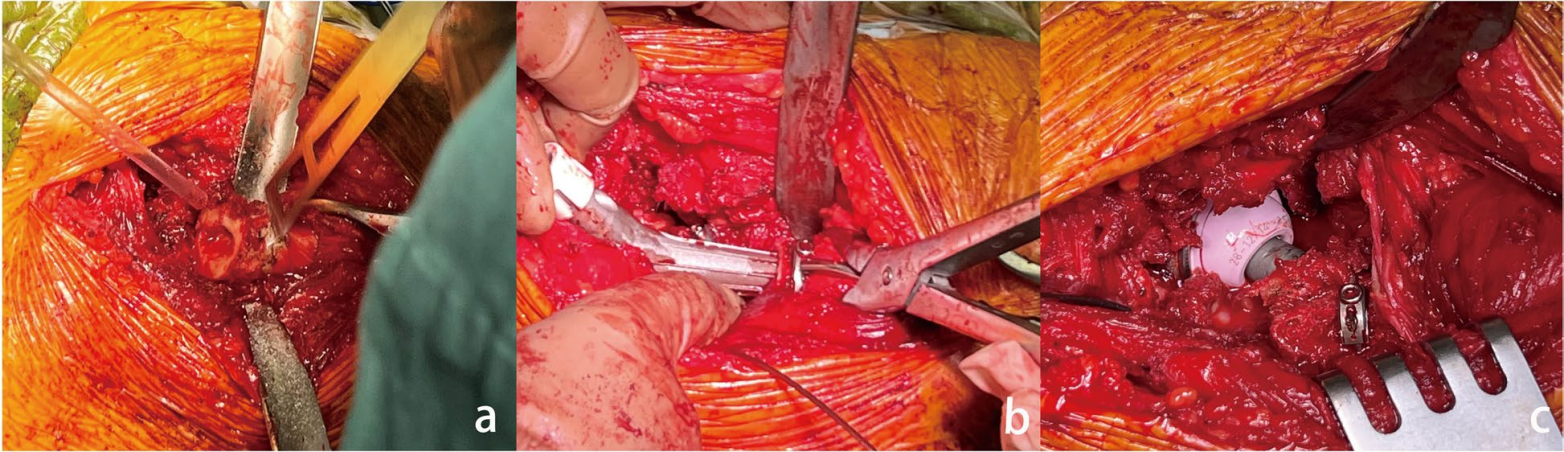
The surgical operation diagram

After installation of the acetabular prosthesis, a test mold of the femoral prosthesis was temporarily implanted into the femoral medullary cavity, and the height of the femur to be amputated was measured based on intraoperative testing. After the surgical bed was folded, the proximal femur was lifted. The assistant maintained the affected limb at extreme adduction and extorsion and rotated the femoral lesser trochanter upward and outward. The attachment of some adductor brevis was visible on the medial surface of the proximal femur. Next, the femoral cortex of the planned osteotomy was exposed by pushing and peeling off the end spots of the adductor brevis. In Fig. 1, The bone was osteotomized perpendicular to the femoral shaft with the protection of musculus vastus lateralis and muscle vastus medialis using two bone levers, followed by the removal of sclerotin planned to be amputated, and The distal and proximal cortices of the femur were tightly fitted by resetting the distal and proximal components of the prosthesis, Rotation was controlled to maintain a certain tension, thus resetting the hip prosthesis.

#### PLA for THA and subtrochanteric osteotomy of the femur

The patient lied on the operating table in a lateral position. A curved incision (approximately 10-12 cm in length) was made in the center of the greater trochanter. The skin, subcutaneous tissues, and fascia were incised layer by layer to expose the intertrochanteric fossa at the posterior margin of the gluteus medius, followed by the cutting of end spots of external rotation muscles. The gluteus medius and sciatic nerve needed to be protected, and the end spots of iliopsoas muscles and gluteus maximus were loosened if necessary. The posterior joint capsule was incised and the femoral neck is amputated 0.5-1.0 cm above the lesser trochanter, followed by the collection of the femoral head. Following the exposure of the acetabulum, the labrum and the syndesmophyte were cleaned, and the acetabulum was filed to the right size number by number for reconstruction. Afterward, acetabular prosthesis and lining were fitted. The femur was exposed by flexion, adduction, and internal rotation of the hip joint and expanded to the appropriate size by reaming the femoral medullary cavity. The joint was reset after installation of the test mold of the prosthesis, subtrochanteric osteotomy, and installation of the S-ROM prosthesis (the same procedure as above).

### Postoperative management

Patients with DAA were given pillows in the popliteal fossa of limbs post-surgery to prevent nerve overstretch. Additionally, patients with PLA were placed in a lying position with the affected limb in a neutral position and at the positions of abducted and flexed hip and knees. Moreover, soft pillows were placed between both limbs to avoid excessive flexion and internal rotation of the hip. Patients in both groups were routinely given antibiotics to prevent infection until 48 h and anticoagulants to prevent thrombosis combined with symptomatic treatment such as multimodal analgesia. Rehabilitation exercises, such as quadriceps isometric contraction exercises and ankle pump exercises, were performed after surgery.

### Postoperative imaging evaluation

The imaging data of patients were obtained from anteroposterior X-ray of bilateral hip joints before surgery and 1 week after surgery. The standard X-ray photography method was as follows: patients lied in the supine position with both lower limbs internally rotated by 15° and Superior margin of pubic symphysis as the center of the photograph. The shape of the obturator and the position of the sacroiliac joint in the preoperative X-ray were referred to exclude the imaging data with a large change in anterior-posterior pelvic inclination and rotation compared with the preoperative period. Dicom files of the X-rays were independently imported into the Mimics Medical 20.0 software by two physicians in a double-blind manner for measurement, followed by the calculation of mean values.

**Fig. 2:**
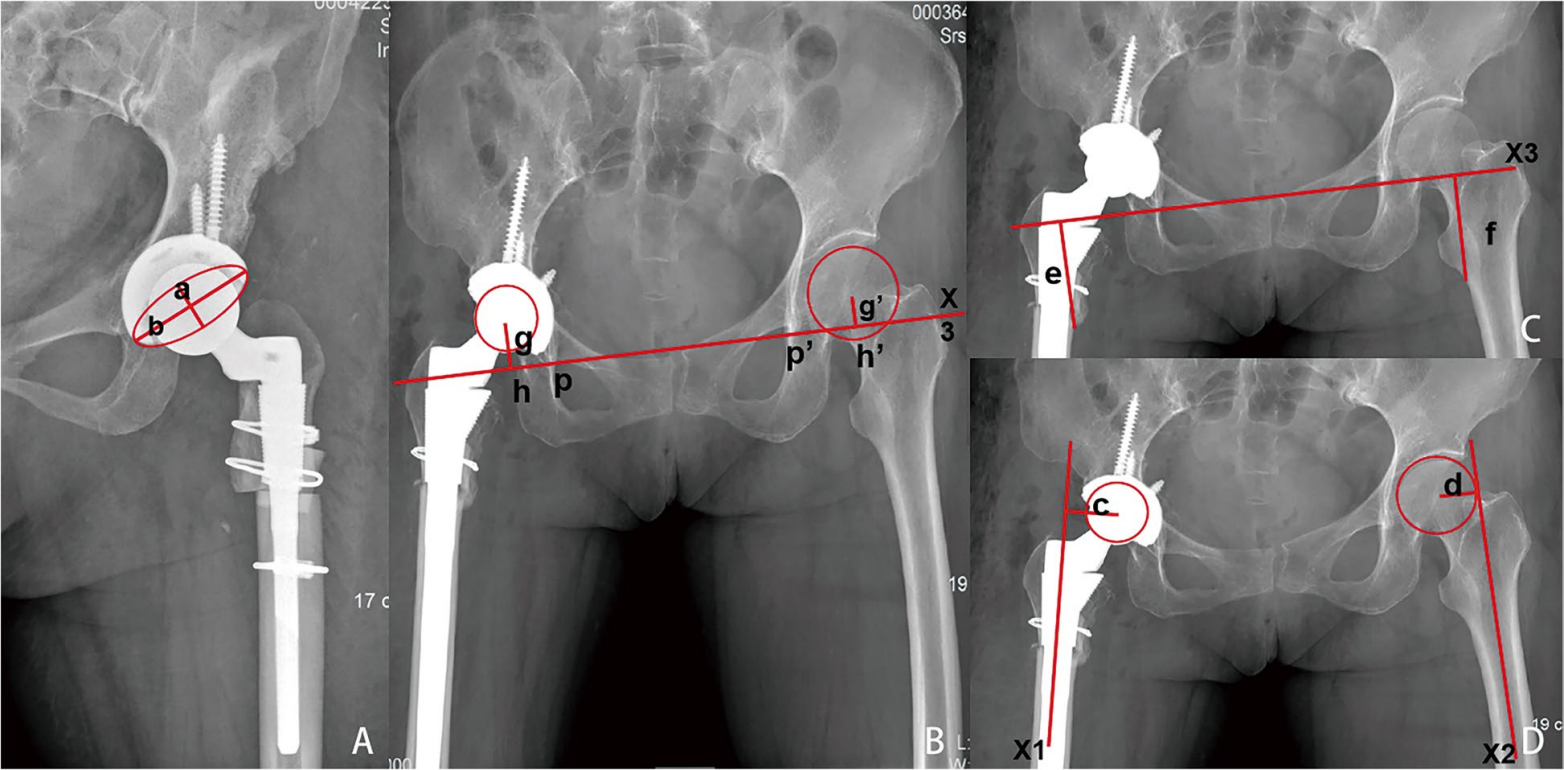
**A** Acetabular anteversion angle: schematic diagram of measuring theacetabular anteversion angle:the elliptical projection of the acetabular cup was drawn to measure the minor axis (**a**) and major axis (**b**) of the elliptical projection. **B** The schematic diagram of measuring the hip rotation center: g and g‘ are the vertical distances from the center of the femoral head on the line of connecting bilateral teardrops (X3); h and h‘ points are the intersection of g and g’ with X3; p and p’ are the lower edge of bilateral teardrops, hp and h’p’ are the horizontal distance of the hip rotation center. **C** Schematic diagram of measuring the leg length discrepancy. **D** Schematic diagram of measuring the femoral eccentric distance

In Fig. 2, the acetabular abduction angle and anteversion angle were referenced to the methods of Lewinnek et al. [14]. The acetabular abduction angle was the angle between the line connecting the bilateral ischial tuberosities and the line connecting the apex of the acetabular cups. The formula to calculate acetabular anteversion angle: acetabular anteversion angle = ARC sine (a/b). The safe area of the acetabular prosthesis was determined according to the methods of Lewinnek et al. [12] and Abdel et al. [15] as the abduction angle of 40 ± 10° and the anteversion angle of 15 ± 10°. The leg length discrepancy was defined as the difference in the vertical distance from the lower margin of the lesser trochanter to the line of connecting bilateral teardrops. The femoral eccentric distance difference was the difference in vertical distance between the center of the femoral head prosthesis on both sides and the extension line of the central axis of the proximal femoral shaft. For hip rotation center measurement, the horizontal difference was identified as the difference in horizontal distance from the center of the acetabular prosthesis on both sides to the extension line of the line connecting bilateral teardrops, while the vertical difference was defined as the difference in vertical distance between the center of the acetabular prosthesis on both sides to the line connecting bilateral teardrops.

### Statistical analysis

SPSS 23.0 software was applied for statistical analysis. The measurement data were expressed as mean ± standard deviation. The independent sample *t*-test was utilized for normally distributed continuous data, and the x^2^ test was employed for the comparison of counting data between groups. The test level α value was taken as two-sided 0.05, and *P* < 0.05 was considered a statistically significant difference.

## Results

No significant differences were observed between the two groups in terms of demographic data (*P* < 0.05) (Table 1).The surgery time in the DAA group (137.35 ± 41.23) was shorter than that in the PLA group (162.36 ± 48.62), whilst the intraoperative blood loss was lower in the DAA group (835.64 ± 349.18)] than in the PLA group (1009.31 ± 600.99) (all *P* > 0.05). the creatine kinase difference was higher in the DAA group (1218.91 ± 1066.62) than in the PLA group (978.23 ± 605.95) (*P* > 0.05). No significant difference was evident between the two group in postoperative Harris score(*P* > 0.05), postoperative LLD (*P* > 0.05). The DAA group (6.50 ± 3.15) exhibited shorter postoperative hospital stay than the PLA group (9.18 ± 4.93), there was statistical significance (*P* = 0.045) (Table 2).

**Table 1:** Comparison of Pre-operative data

Group	DAA group (*n* = 20)	PLA group (*n* = 22)	t/x^2^	*P*-Value
Age (years)	52.95 ± 14.76	48.91 ± 16.84	0.823	0.415
BMI (kg/m^2^)	24.09 ± 3.46	23.16 ± 4.52	0.748	0.459
Gender (male/femal)	6/14	4/18	0.807	0.369
Operations side (left/right)	15/5	10/12	3.796	0.051
Pre-op Harris score	52.20 ± 5.13	53.91 ± 4.67	−1.113	0.265

**Table 2:** Comparison of Post-operative data

Group	DAA group (*n* = 20)	PLA group (*n* = 22)	t/x^2^	*P*-Value
Operation time (min)	137.35 ± 41.23	162.36 ± 48.62	−1.789	0.081
Post-op LOH (days)	6.50 ± 3.15	9.18 ± 4.93	−2.075	0.045*
Intraoperative blood loss (mL)	835.64 ± 349.18	1009.31 ± 600.99	−1.130	0.265
Creatine kinase (D1–0) (U/L)	967.60 ± 570.83	978.33 ± 605.95	−0.058	0.954
Post-op Harris score	88.00 ± 3.97	87.09 ± 2.11	0.938	0.354
Post-op LLD (mm)	5.45 ± 4.37	7.23 ± 4.55	−1.290	0.205
The number of osteotomies	9	13	0.834	0.361

*LOH* length of hospitalization, *post-op* post-operative, *LLD* leg length discrepancy, *D1–0* the difference of creatine kinase between the 1 day after surgery and before surgery

*Significant difference

Among the complications in both groups at follow-up, there was one case with a proximal femoral split fracture in the DAA group and one case with a distal femoral split fracture in the PLA group. Moreover, the rate of intraoperative fracture was higher in the DAA group than in the PLA group (5.00% vs 4.45%, *P* > 0.05). In addition, the rate of postoperative venous thrombosis in the lower limbs was higher in the DAA group than in the PLA group (10.00% vs 4.45%, *P* > 0.05). Meanwhile, there were three cases of femoral nerve paralysis and one case of postoperative dislocation in the PLA group. All of the cases with femoral nerve paralysis took neurotrophic drugs for about 1 week, and the nerve paralysis symptoms completely disappeared 6 months after surgery. Additionally, the percentage of postoperative difference in the length of both lower limbs > 10 mm was 15% in the DAA group and 18% in the PLA group, without a statistically significant difference (*P* > 0.05). The incidence of complications in the DAA group was 30%, which was higher than that in the PLA group (45.00%) (Table 3).

**Table 3:** Comparison of the complications in DAA group and PLA group

Complications	DAA group (*n* = 20)	PLA group (*n* = 22)	x^2^	*P*-Value
Intraoperative fracture	1 (5.00%)	1 (4.45%)	–	1.000
Venous thrombosis in the lower limbs	2 (10.00%)	1 (4.45%)	–	0.598
Anterior thigh skin numbness	0	3 (13.64%)	–	0.233
Postoperative dislocation	0	1 (4.45%)	–	1.000
Unacceptable LLD (> 10 mm)	3 (15%)	4 (18%)	–	1.000

The acetabular abduction and anteversion angles in the DAA and PLA groups were (44.96 ± 4.75 vs 43.85 ± 4.92) and (18.97 ± 4.16 vs 16.57 ± 5.55), respectively (*P* > 0.05), and The DAA group displayed 85.00% of both the abduction and anteversion angles within the safe area, higher than 81.82% of the PLA group (*P* > 0.05). The horizontal differences in hip rotation center of the DAA and PLA groups were (4.34 ± 3.11 vs 6.99 ± 4.87), there was statistical significance (*P* = 0.044). The horizontal differences and the femoral eccentric distance difference was (4.67 ± 3.96 vs 4.15 ± 3.53) and (5.37 ± 3.71 vs 6.80 ± 5.08), respectively (*P* > 0.05) (Table 4).

**Table 4:** Comparison of postoperative prosthesis placement angle between DAA group and PLA group

Group	DAA group	PLA group	t/x^2^	*P*-Value
Acetabular abduction angle (°)	44.96 ± 4.75	43.85 ± 4.92	0.737	0.465
Acetabular anteversion angle (°)	18.97 ± 4.16	16.57 ± 5.55	1.575	0.123
the safe area the safe accuracy (%)	85.00%	81.82%	–	1.000
The difference of rotation center				
Horizontal differences (mm)	4.34 ± 3.11	6.99 ± 4.87	−2.081	0.044*
Vertical differences (mm)	4.67 ± 3.96	4.15 ± 3.53	0.444	0.659
Femoral eccentric distance difference (mm)	5.37 ± 3.71	6.80 ± 5.08	−1.036	0.307

*Significant difference

## Discussion

Most studies of DDH have elucidated that THA for high-dislocated DDH using PLA can achieve favorable clinical results and is recognized by the overwhelming majority of clinicians [13, 16]. On the one hand, the true intermuscular plane is used for DAA, which can fully preserve piriformis muscles, external rotation muscles, gluteus medius, and other muscles, thus showing the advantages of less damage to soft tissues, less bleeding, and no contraindicated postoperative position. On the other hand, an interneural plane is utilized for DAA to reduce nerve damage and stabilize abductor muscles more quickly after surgery, which is conducive to rapid rehabilitation. DAA is gradually being applied in hip surgeries compared to the traditional surgical approaches, but it has rarely been reported for more complex primary cases, especially for the treatment of severe DDH. The results of this study manifested that the efficacy of THA via DAA in high-dislocated DDH was similar to that of THA via PLA in terms of bleeding reduction, tissue damage, and limb length discrepancy shortening, DAA-THA could shorten the postoperative length of hospitalization, proving that DAA is a feasible surgical approach.

In patients with Crowe III-IV DDH, THA is complicated because of the extensive pathomorphological alterations of the acetabulum and femur, as well as the diversity and youth of the patients. Therefore it is difficult to reconstruct the acetabulum, correct the femoral anteversion angle and high valgus neck-shaft angle, and restore the center of rotation, which is challenging for prosthesis placement [17]. Liu et al. [18] conducted a study of the postoperative X-ray analysis of patients with Crowe III-IV DDH undergoing DAA (23 cases) and PLA (47 cases), which elaborated no significant difference in the placement of the two components, except for the elevated acetabular anteversion angle in the DAA group. This study also depicted difference in the angle of prosthesis placement following the two approaches for THA, indicating that the DAA approache exerted beneficial effect on prosthesis position. The reasons for this are: (1) Reconstruction of the acetabulum is particularly essential. Due to a large amount of soft tissue contracture in high-dislocated DDH, the true acetabulum is covered by soft tissues, and thereby it is difficult to find the true acetabulum. In this study, the surgeon located the true acetabulum at the superior margin of the obturator, loosened the soft tissues, and installed the acetabular prosthesis with the transverse ligament as a marker for acetabular reaming and the Harris fossa and acetabular notch as a marker to determine the center of the acetabulum. (2) The modular femoral stem prosthesis S-ROM was adopted, in which the mutually independent design of the proximal cuff and stem can adjust the femoral anteversion angle and restore the offset distance in a wider range. In addition, the distal golden fork and metal ridge induce anti-rotational stabilization during osteotomy, and the sleeve is both porous and hydroxyapatite coating to convert shear stresses into compression forces at the sleeve-bone interface. A study by Noble et al. [2] on the three-dimensional shape of the femur in DDH uncovered that the femoral anteversion angle increased by anywhere from 5° to 16° with enhancing subluxation. (3) Combined with intraoperative fluoroscopy, it is easier to locate accurately and safely.

The most prevalent complications following high-dislocated DDH consist of postoperative dislocation, intraoperative femoral fracture, nerve paralysis, and non-union of the femoral osteotomy [16, 19]. According to the literature, the incidence of femoral fractures can reach 0.1 to 27.8% during THA [20]. Transverse osteotomy with prophylactic fixation of the distal fragment with the S-ROM prosthesis is a safe method, and the one-stage procedure does not increase the risk of femur fracture. In our research, we observed that fractures still occurred, mainly related to femoral reaming. Firstly, during distal femoral reaming, a straight reaming file is used to grind the femur to the same diameter as the stem. When the distal reaming is too excessive, the cortical bone is thin and the implanted stem is too large to be prone to distal fracture. If a long curved stem is employed, grinding diameters of more than 1.5 mm can avoid fracture during stem implantation. Secondly, when proximal reaming is performed, the mark of the center of rotation is determined by the greater trochanter of the femur, the mark on the handle of the conical file corresponds to the greater trochanter. Furthermore, the reaming is stopped when the reaming is felt to be tight, followed by femoral calcar reaming. Excessive proximal reaming and an oversized implanted cuff lead to fracture of the proximal femur. When a femoral prosthesis is implanted, a varus position of the implanted stem can also cause the caudal end of the stem to impinge on the anterolateral femoral cortex, thus resulting in a fracture.

Postoperative dislocation after DDH is one of the common surgical complications, with a reported dislocation rate of 1.6-16.6% [17]. In this study, only one case of postoperative dislocation was observed, and it was documented that the surgical approach did not correlate to the postoperative dislocation rate. However, there was a limitation of a small sample size, so the results are not representative. The surgeons believe that the reduction of the postoperative dislocation rate lies in two aspects. One aspect is the placement of the prosthesis. Specifically, the restoration of the rotation center of the hip joint and the correct positioning of the acetabulum are closely related to the reduction of the dislocation rate. It is currently accepted that the prosthesis position is within the Lewinnek functional safe area. Furthermore, there are some studies for further discussion. For instance, Yetkin et al. used a multivariate analysis to illustrate that the risk of postoperative hip dislocation was 2.62 times higher in patients with an acetabular abduction angle above the range of 35°-45° and 2.90 times higher in those with a high hip center [21]. Zhou et al. noted that the use of a larger femoral head and the improvement of abductor strength helped to reduce the incidence of postoperative dislocation [22]. The other aspect is to loosen the soft tissues in place. Abductor muscles are very weak in high-dislocated DDH, and the complex of abductor muscles should be protected intraoperatively. Compared with PLA, the DAA approach can preserve the gluteus medius and piriformis muscles and improve the postoperative muscle strength of hip flexors and adductor muscles more rapidly.

Postoperative nerve injuries after THA include lateral femoral cutaneous nerve injury, sciatic nerve injury, and common peroneal nerve injury, whose incidence mainly ranges from 0.1 to 7.6% [23]. Moreover, these injuries are triggered by direct injury or excessive limb lengthening during surgery. The use of osteotomy and limb lengthening of no more than 3-4 cm can obviously diminish the risk of nerve injury in high-dislocated DDH. Femoral nerve injury is mainly manifested by abnormal sensation in the anterolateral thigh and medial lower leg with a low incidence, which was previously common in traditional surgical approaches. In this study, only femoral nerve paralysis was found, indicating that it should be taken seriously in the complications of DDH, especially in PLA. The causes of paralysis may be correlated with retractor placement compression, hematoma formation, improper traction, ischemia, or thermal injury. Therefore, care should be taken to avoid injuries to the femoral nerve by the tip of retractors when placing retractors at the anterior margin of the acetabulum. Intriguingly, a recent study on the proximity of neurovascular structures unraveled that the anterior inferior iliac spine was the safest location for an anterior acetabular retractor [24]. Regarding the prognosis of femoral nerve paralysis, it has been reported in the literature that femoral nerve paralysis has better prospects for functional recovery compared to other nerve paralyses after THA with almost complete recovery after only gentle exercise [25–28].

This study is a retrospective nonrandomized design with the limitations of small sample size and no interim follow-up clinical data. In addition, CT is the gold standard for the measurement of acetabular anteversion angle but is not a routine postoperative follow-up examination. In this study, although the anteversion angle of the prosthesis was measured based on X-rays was subject to some error, which was proved to be reliable and its accuracy is close to the CT [29–33]. Addressing these limitations will require a large number of multicenter, multisample, and prospective randomized controlled studies to explore the exact value of THA via different surgical approaches for the efficacy of Crowe III and IV DDH. Nevertheless, this study has some advantages. First, all patients were uniformly operated on and followed up by the same surgeon, with consistent surgical procedures and follow-up plans, which reduces the error of subjective bias of multiple surgeons in comparative studies. Second, this study encompasses an accurate review of postoperative X-ray images (especially the center of rotation and femoral eccentric distance) for DAA and PLA. Third, this is also the first paper to our knowledge of a comparative study of both DAA and PLA procedures using the S-ROM prosthesis.

## Supplementary Information

**Additional file 1 .**

## Data Availability

The datasets used and/or analyzed during the current study are available from the corresponding author on reasonable request; please contact the corresponding author, Dr. Feng. Administrative permission was received from The Third Clinical Medical College, Fujian Medical University (No. 47, Shangteng Road, Cangshan District, Fuzhou, China) to access the medical records.

## Abbreviations

DDH: Developmental dysplasia of the hip
PLA: Posterior lateral approach
DAA: Direct anterior approach
THA: Total hip arthroplasty
LOH: Length of hospitalization
post-op: post-operative
LLD: Leg length discrepancy
D1–0: The difference of creatine kinase between the 1 day after surgery and before surgery
