# Correction: Direct anterior versus posteriorlateral approachs for clinical outcomes after total hip arthroplasty in the treatment of severe DDH

**DOI:** 10.1186/s12891-022-06011-3

**Published:** 2022-11-30

**Authors:** Yiping Lan, Eryou Feng, Bei Lin, Zhiming Lu, Feitai Lin, Yan Weng

**Affiliations:** 1 The Third Clinical Medical College, Fujian Medical University, No. 47, Shangteng Road, Cangshan District, Fuzhou, China; 2 Fujian University of Traditional Chinese Medicine, Fuzhou, China

**Correction: BMC Musculoskelet Disord 23, 958 (2022)**

10.1186/s12891-022-05759-y

Following publication of the original article [1], one of the authors reported that his byline is not correct. The author’s family name “Lin” has a missing “n” in the published article, this error was introduced during production process. The author’s complete name should be “Bei Lin“ instead of “Bei Li“.
