# Supplementary figures and images for: Direct anterior versus posteriorlateral approachs for clinical outcomes after total hip arthroplasty in the treatment of severe DDH
Source: BMC Musculoskelet Disord. 2022 Nov 5;23:958. doi: 10.1186/s12891-022-05759-y (PMC9636645; doi:10.1186/s12891-022-05759-y)

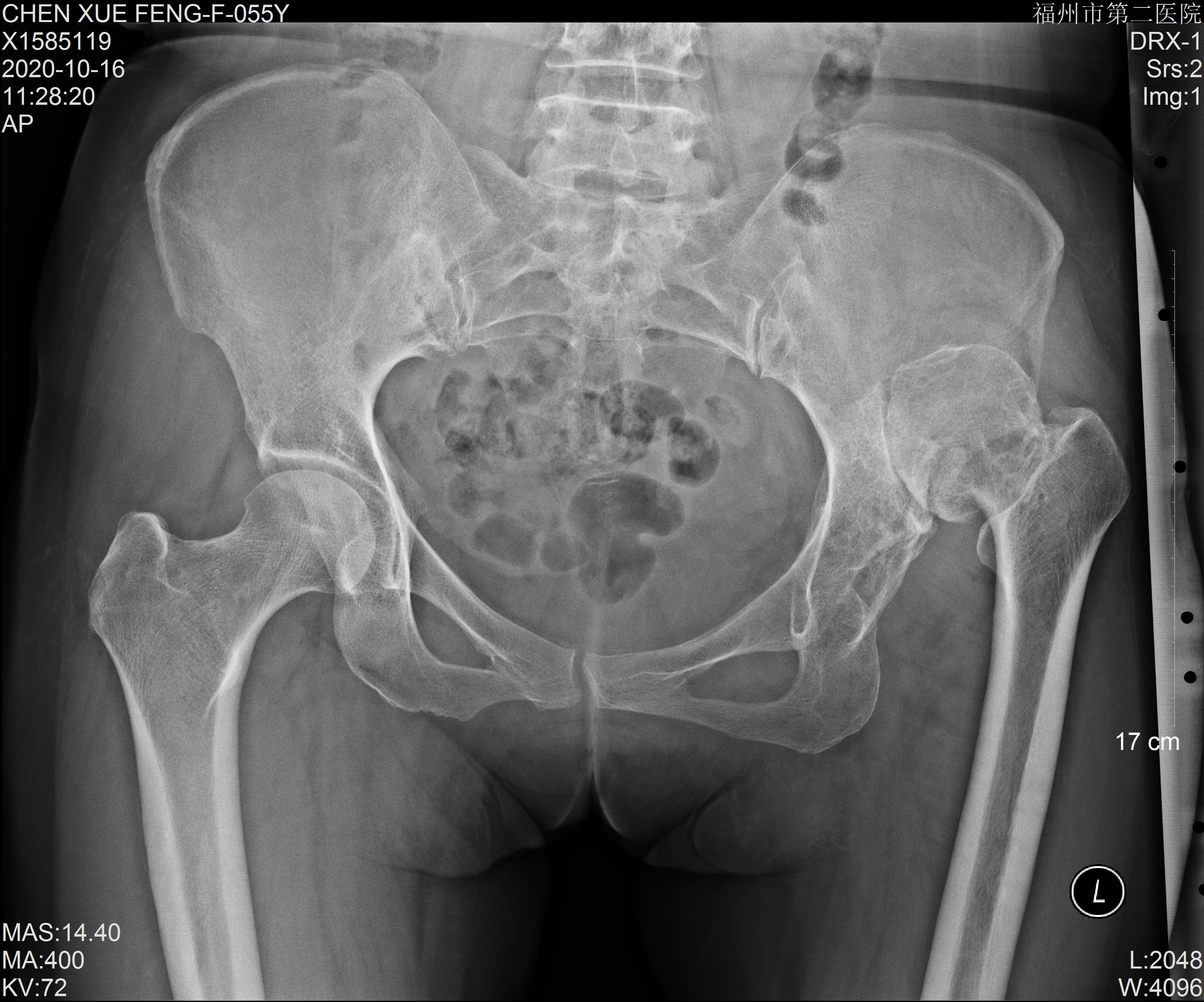

Supplement: Supplementary file 1 — Additional file 1 . [file 12891_2022_5759_MOESM1_ESM.zip › file 1/fig.1.jpg]

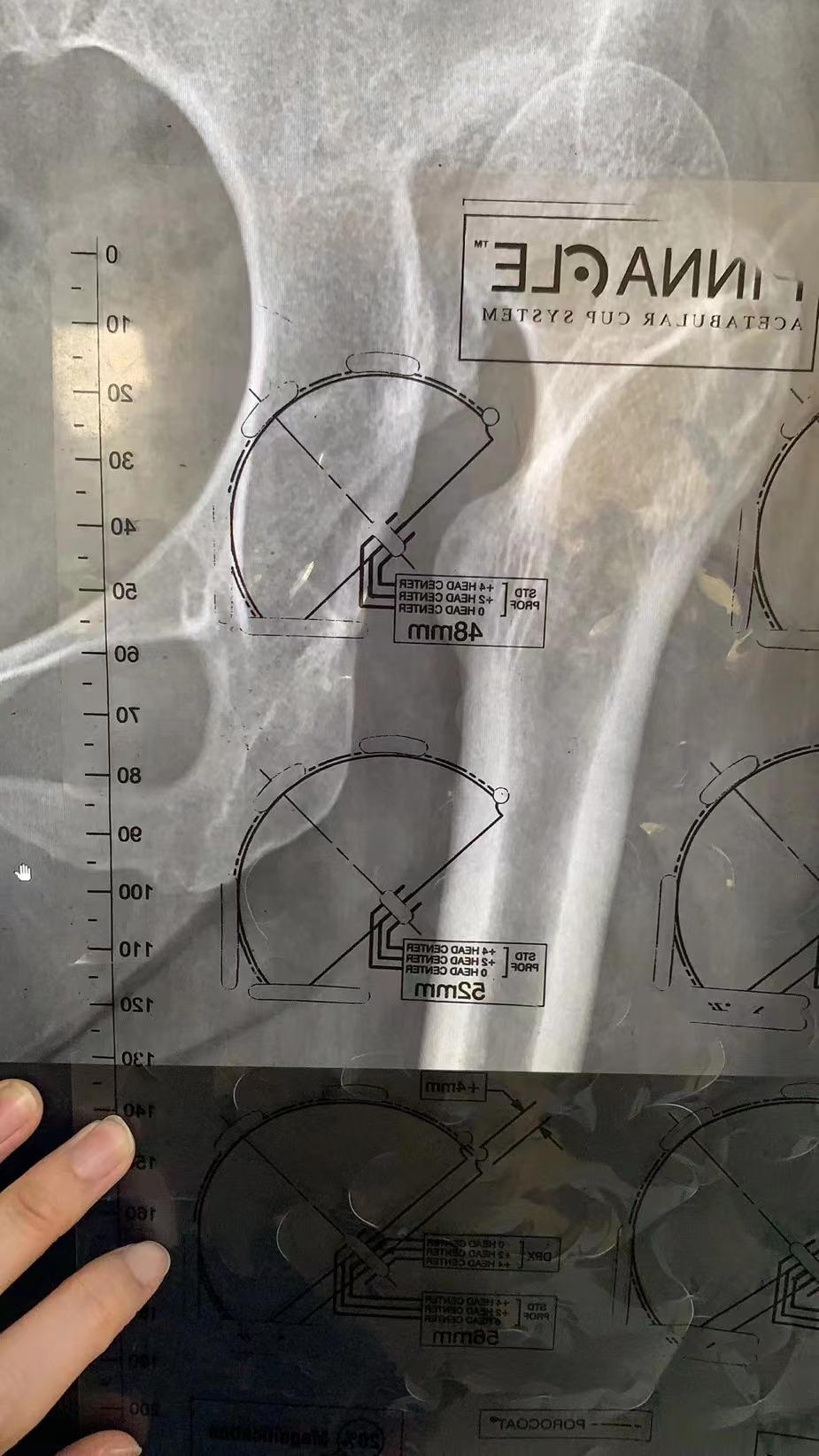

Supplement: Supplementary file 1 — Additional file 1 . [file 12891_2022_5759_MOESM1_ESM.zip › file 1/fig.2.jpg]

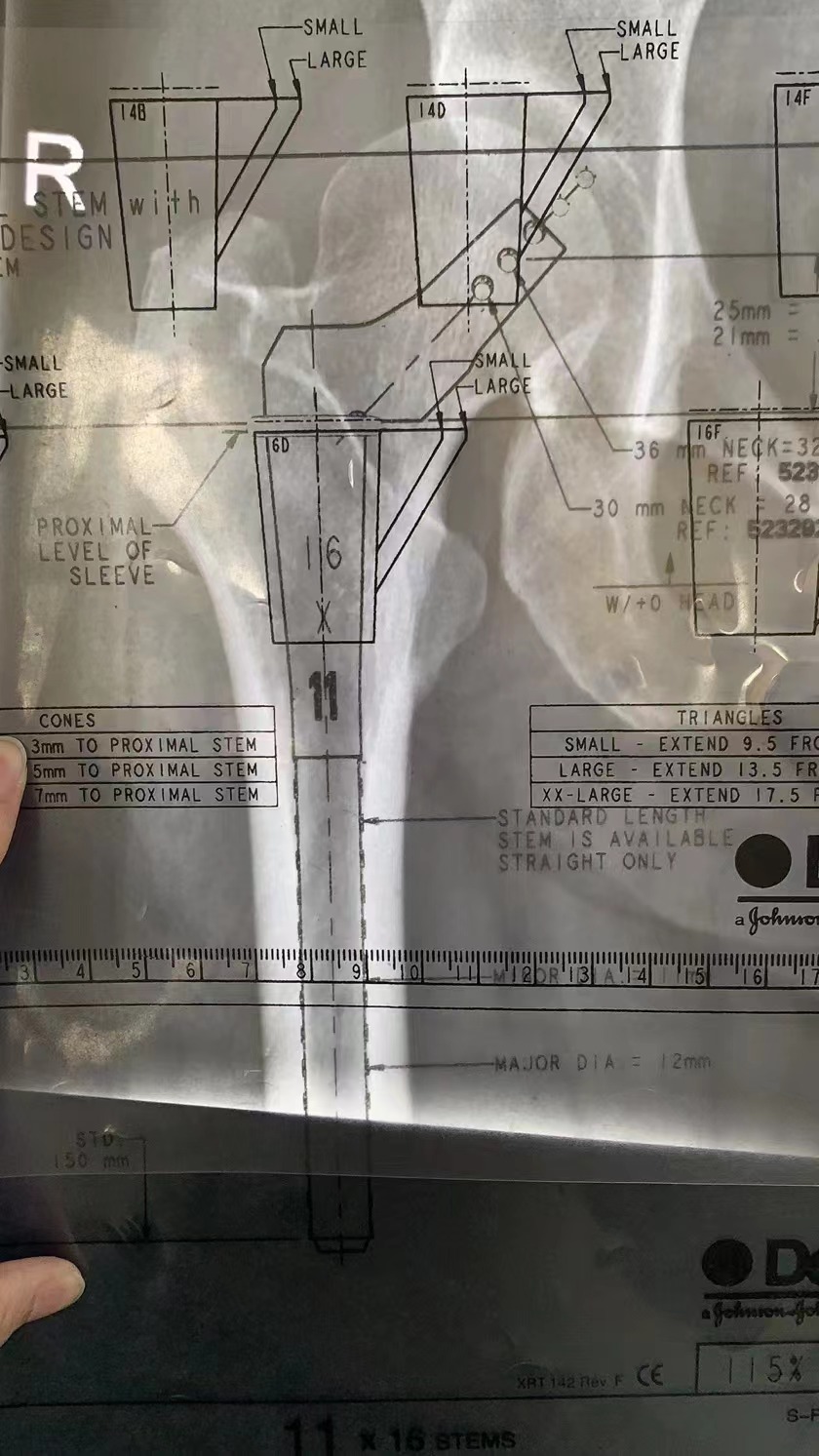

Supplement: Supplementary file 1 — Additional file 1 . [file 12891_2022_5759_MOESM1_ESM.zip › file 1/fig.4.jpg]

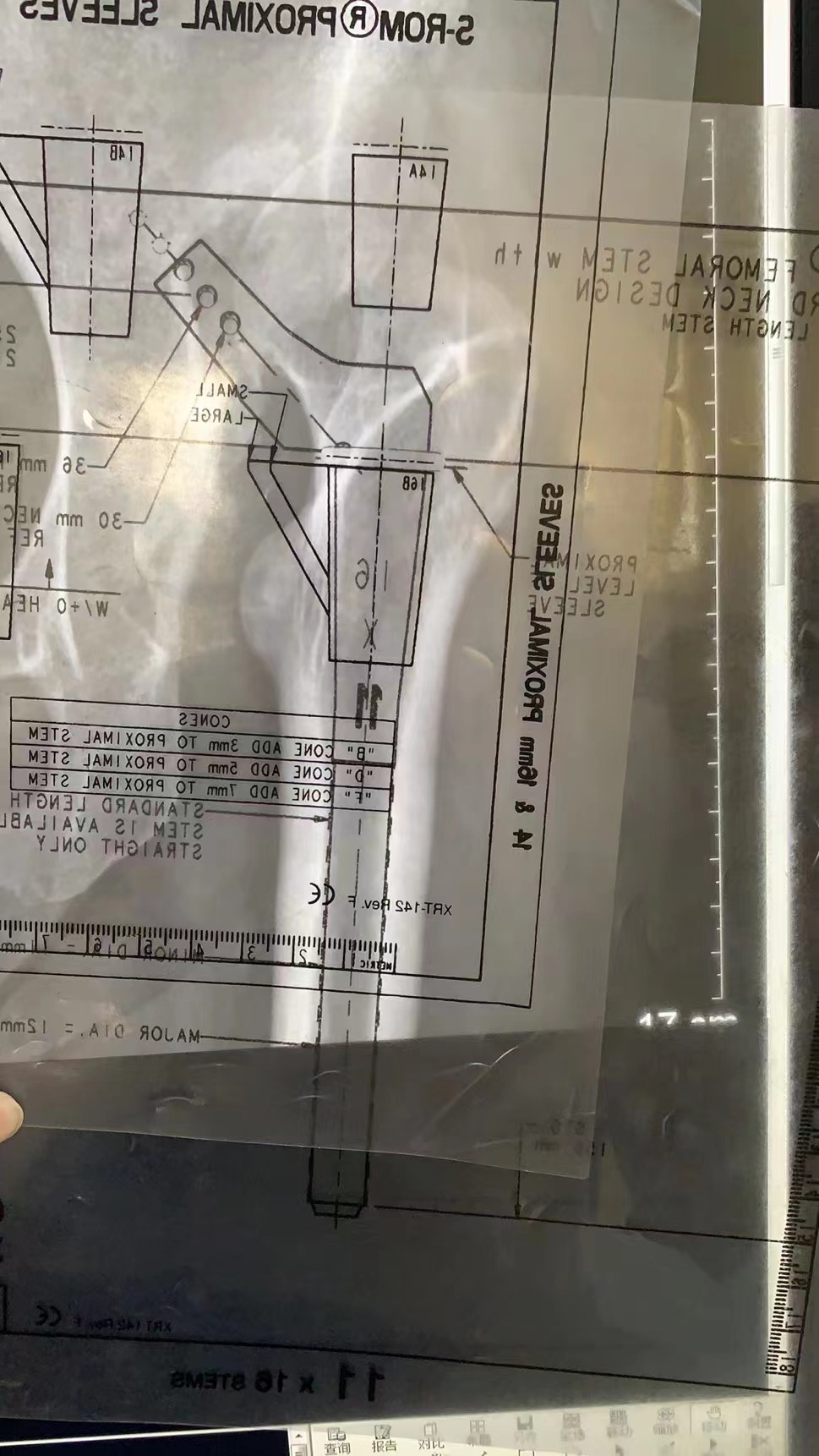

Supplement: Supplementary file 1 — Additional file 1 . [file 12891_2022_5759_MOESM1_ESM.zip › file 1/fig3.jpg]
